# In Vitro, Ex Vivo and Clinical Trial of Brightening Serum Using a Novel Delivery System on Subjects With Moderate to Severe Dyschromia

**DOI:** 10.1111/jocd.70686

**Published:** 2026-02-05

**Authors:** Ashish C. Bhatia, Arisa Ortiz, Swati Kannan, Amir Moradi, Tina Fleck, Robert Love, Stacy Osborne

**Affiliations:** ^1^ Northwestern University Feinberg School of Medicine Chicago Illinois USA; ^2^ Department of Dermatology UC San Diego Health San Diego California USA; ^3^ Moradi MD/Private Practice Vista California USA; ^4^ Pacific Clinical Innovations/Clinical Research Site Vista California USA; ^5^ Ourself Carlsbad California USA

**Keywords:** cosmetic, dyschromia, photodamage, skin of color, skin tone unevenness, topical delivery

## Abstract

**Background/Aims:**

Pigmentary problems are common and can arise from sun exposure, skin inflammation, changes in hormones, and certain medications. Because they are visible, these pigmentary problems can be very distressing for individuals, and they are often challenging to manage. Three studies were conducted, ex vivo, in vitro, and in vivo.

**Subjects/Methods:**

An in vitro study of brightening/lightening in skin models; an ex vivo skin study using Raman spectroscopy; and a 12‐week clinical study of a novel complexion brightening serum with Tiered‐Release Vesicle (CBS‐TRV) delivery technology in adults with moderate to severe photodamage and uneven skin tone. Assessments included clinical grading, melanin and erythema measurements by Mexameter, digital imaging, and a patient self‐assessment questionnaire. Standard safety and tolerability assessments were also performed.

**Results:**

The in vitro 3D skin study showed that a 2 μL/cm^2^ application of CBS‐TRV (the recommended and clinical application volume) slowed melanin production by 37.5% compared to untreated tissues after 2 weeks. Furthermore, Raman spectroscopy provided evidence for tranexamic acid penetration into the epidermis of ex vivo human skin. Clinical grading revealed significant improvements at the majority of visits, which were accompanied by improvements of erythema index and melanin index on mexameter. In addition, subjects provided positive feedback regarding the clinical results as well as the formulation's aesthetics. Finally, the CBS‐TRV product was safe and well tolerated.

**Conclusions:**

The multiple active ingredients in CBS‐TRV target melanogenesis in a variety of ways, and both in vitro and clinical studies demonstrated significant improvements in skin tone and pigmentation.

## 1 Introduction

Facial hyperpigmentation is common and can negatively impact individuals' quality of life and psychological well‐being [1, 2]. These factors are particularly important for aging individuals and those with skin of color, who require careful formulation strategies to minimize irritation and rebound pigmentation. Despite some effective treatments for hyperpigmentation, management is challenging and relies heavily on photoprotection strategies [2]. Common medical approaches include hydroquinone and procedures such as chemical peels and laser treatments; however, these may be associated with high costs or adverse side effects [2]. Given the demand for management strategies perceived to be safer than prescription, it is important for clinicians to be aware of such products, their active ingredients, and their efficacy and safety.

The term hyperpigmentation describes a condition where patches of skin become darker (with or without redness) than surrounding skin due to an excess production and deposition of melanin. There are internal factors (aging, hormones), environmental factors (sun exposure, dry climates, heat, pollutants, physical injury/trauma), and medical disorders that can contribute to skin tone unevenness along with superficial dry scaly skin, wrinkles, small capillaries or diffuse and mottled brown patches [3]. In most forms of acquired hyperpigmentation, extrinsic stimuli such as UV radiation or inflammation induce keratinocytes and fibroblasts to release local signaling factors that stimulate melanogenesis in melanocytes; the pigment is then transferred to keratinocytes as a protective barrier. However, in conditions such as melasma, drug‐induced pigmentation, or certain genetic disorders, intrinsic or systemic factors may also drive melanogenesis independent of keratinocyte signaling.

We tested a novel complexion brightening serum (CBS) that uses Tiered‐Release Vesicle (TRV) technology for enhanced topical delivery of several key molecules. The multi‐component formulation was designed to enhance penetration of various sized molecules through the stratum corneum (SC) for optimized activity in the epidermis [4]. The CBS‐TRV product achieves significant skin brightening and skin tone evenness. The active ingredients in CBS‐TRV affect the melanin synthesis pathway in several ways: (1) A novel, patented peptide analog of melanocyte stimulating hormone a‐MSH, here designated MI9, competes with a‐MSH for binding to the MC1 receptor on melanocytes, thus blocking the signal for melanin production [5, 6]. Given the molecular weight of MI9 (> 1000 Da), it is not expected to penetrate the SC [7]; however, TRV technology enables entry of fluorescent labeled peptides of similar size and charge [4]. (2) 4‐butylresorcinol (4BR) reduces the synthesis of melanin within melanocytes through inhibition of critical enzymes such as tyrosinase and tyrosinase‐related protein‐1 [8, 9]. (3) Niacinamide can slow the transfer of pigment from melanocytes to keratinocytes [10, 11]. (4) Tranexamic acid (TXA) hinders the conversion of dermal plasminogen to active plasmin, ultimately lowering the production of inflammatory prostaglandins that stimulate melanocytes to produce melanin [12, 13, 14]. Attempts at topical application of TXA for treating melasma have been reported [15, 16, 17], but the topical route is challenging due to poor permeability through the SC [18, 19, 20]. The combination of TRV technology and supplementation with an ester form of TXA allows the CBS‐TRV formulation to deliver TXA into the SC and epidermis, where it can positively impact keratinocyte cells. (5) A vitamin C derivative, aminopropyl ascorbyl phosphate, provides anti‐antioxidant activity [12]. (6) Retinyl linoleate, an ester linkage of retinol (Vitamin A) and linoleic acid, slows melanin production and increases epidermal turnover of pigmented cells [21, 22].

In the present study, we assess the effects of topical CBS‐TRV in clinical trials over a period of 12 weeks, and demonstrate significant improvement in skin tone and pigmentation.

## 2 Materials and Methods

### 2.1 Materials

Peptides were made using solid phase synthesis and purified to 95% by high performance liquid chromatography (HPLC), followed by sequence confirmation and storage at −20°C as a lyophilized powder. The activity of proprietary peptide MI9 was confirmed in a cell‐based system [Eurofins Panlabs, Taiwan]. Tranexamic acid (TXA) was obtained from Spec‐Chem Industry Inc., and Cetyl Tranexamate Mesylate was supplied by Actera Ingredients. The latter is expected to convert into TXA within the skin [23], and both were included in the formulation to increase the probability of final TXA delivery into viable layers.

### 2.2 Ex Vivo Study

The presence of TXA in ex vivo human skin explants after topical CBS‐TRV was assessed by RAMAN spectroscopy at Eurofins BIO‐EC (France). Fifteen human skin explants of an average diameter of 11 mm (±1 mm) were prepared on an abdominoplasty coming from a 49‐year‐old woman with a phototype II according to Fitzpatrick skin color classification. The explants were kept in survival in BEM culture medium at 37°C in a humid, 5% CO_2_ atmosphere. On Day 0, products were topically applied on the basis of 5 μL per 1 cm^2^ explant. The control explants did not receive any treatment. On Day 1 (24 h after product application), 3 explants from each batch were collected, cut in two halves and stored at −80°C. Half frozen explants underwent RAMAN spectroscopy analysis. For each explant, 3 skin tissue sections (20 μm‐thick) were deposited on a CaF2 specific support for Raman imaging analysis. One Raman image was recorded for each explant, and the final results averaged. An average signal of the control batch (background skin signal) was subtracted from profiles observed for the test batches.

### 2.3 In Vitro Study

MelanoDerm (MatTek Corporation, Ashland, MA) tissues were used to assess the formulation's effectiveness at reducing natural melanin production during topical exposure using EPI‐100‐NMM‐113 media. MEL‐300‐B tissues in triplicate were exposed to a test volume (2 μL), similar to the coverage per square centimeter of skin in the clinical trials. Topical 2% Kojic acid (25 μL; *n* = 3 tissues) served as a positive control (PC) for skin lightening. Some tissues (*n* = 3) remained untreated to serve as a negative control (NC). MelanoDerm tissues were treated for a total of 14 days with the PC and test material treated tissues dosed topically on Days 0, 2, 5, 7, 9, and 12. Tissues were analyzed on days 0, 5, 9, and 14 for surface reflectance (L*D65) as a measure of skin pigmentation using a Konica Minolta Color Spectrophotometer (CM‐700d). Finally, on day 14, tissues were evaluated for tissue viability using an alamarBlue assay and *n* = 3 tissues per group were collected for melanin quantification.

### 2.4 Clinical Trial

This was a 12‐week, open label study evaluating tolerability and effectiveness of CBS‐TRV (Ourself, Carlsbad, CA, USA) plus Daily Renewal Cream (Ourself), cleanser and sunscreen SPF 45, to improve skin tone unevenness in adults with moderate‐to‐severe facial dyschromia. Products were applied twice daily (except sunscreen, applied in the morning and as needed). The study complied with Good Clinical Practice, and all subjects gave written informed consent, including permission for scientific and educational use of photographs. The protocol, informed consent, and all addenda were reviewed and approved by the Advarra Central IRB (Ontario, Canada).

#### 2.4.1 Patient Population

Eligibility criteria included adults aged 22–75 years, inclusive of all genders and all Fitzpatrick skin phototypes. According to the study protocol, a minimum of six subjects were to have Fitzpatrick phototypes V–VI. All subjects had moderate to severe photodamage and uneven skin tone (score of 4–9 according to a modified Griffiths scale [24] where 0 = none and 9 = severe). Subjects had no facial treatments within the previous 6 months and agreed to avoid them during the study. Primary exclusion criteria included a history of skin cancer within the past 5 years; current use of acne medications; isotretinoin use within the past 6 months; hydroquinone use within the past 12 months; use of prescription‐strength skin‐lightening products; use of topical or systemic products known to affect skin aging or dyschromia within the past 3 months; known intolerance to retinoids; and steroid use within the past 4 weeks.

#### 2.4.2 Clinical Assessments

Efficacy was assessed at baseline and at weeks 2, 4, 8, and 12. Assessments included clinical grading, melanin and erythema measurements obtained with a narrow‐band reflectance spectrophotometer (Mexameter MX18, Courage + Khazaka electronic GmbH, Köln, Germany), and digital imaging with the VISIA CR system (Canfield Scientific Inc., Parsippany, NJ). Standardized digital photographs were taken of each subject's face (right, center, and left views) under standardized illumination (cross‐polarized, parallel‐polarized, and UV lighting). Clinical grading included skin smoothness (tactile on the cheeks), skin smoothness (visual), appearance of pores, skin tone evenness, and global facial radiance/luminosity/brightness. In addition, subjects completed a self‐assessment questionnaire. Standard safety and tolerability assessments were performed, including objective irritation (erythema, edema, dryness, and scaling), subjective irritation (burning, stinging, and itching), and collection of adverse events.

#### 2.4.3 Statistical Methodology

Demographic data, baseline characteristics, and continuous variables were summarized using descriptive statistics. Frequencies and percentages were reported for categorical variables. For each evaluation parameter, mean percent change from baseline and the percentage of subjects who improved or worsened were calculated at applicable post‐baseline timepoints. Questionnaire responses were tabulated and the frequencies and percentages were summarized. Wilcoxon rank test was used to analyze clinical grading, tolerability, and quality of life questionnaire parameters. Paired *t* test was used for mexameter results. All statistical tests were 2‐sided at significance level alpha = 0.05 unless specified otherwise. Statistical analyses were performed using SAS software version 9.4 (SAS Statistical Institute, Cary, North Carolina).

## 3 Results

### 3.1 Ex Vivo Study

**FIGURE 1:**
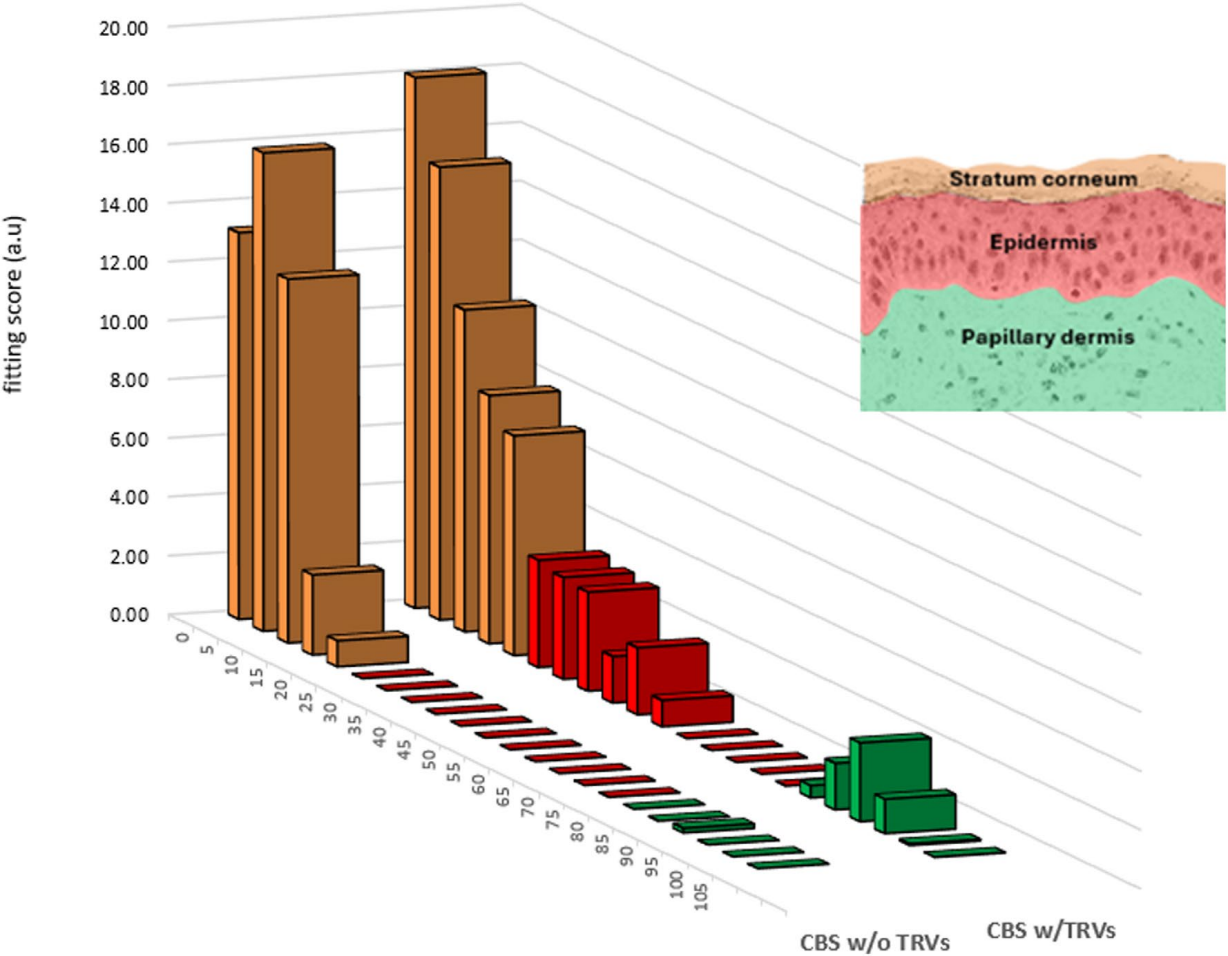
Semi‐quantitative representation of Raman signal for TXA within ex vivo skin after 24 h application of a CBS formulation, with or without TRV formation, and as a function of skin depth (μm). Layers of skin are colored according to stratum corneum (orange), epidermis (red), and papillary dermis (green).

After topical application of CBS‐TRV formulation to ex vivo skin for 24 h, Raman analysis of skin sections showed significant TXA penetration into the SC, with small amounts detected up to 40–45 μm depth (corresponding to the epidermis) and trace amounts up to 80–90 μm depth (corresponding to the papillary dermis); see Figure 1. Penetration beyond the SC and into the epidermis was not seen with a simple mixture of the raw materials used for CBS‐TRV (which includes penetration enhancers and simple liposomes but not multilamellar TRVs; referred to here as CBS w/o TRVs). Thus, effective delivery requires complete formation of the TRVs [4].

### 3.2 In Vitro Study

**FIGURE 2:**
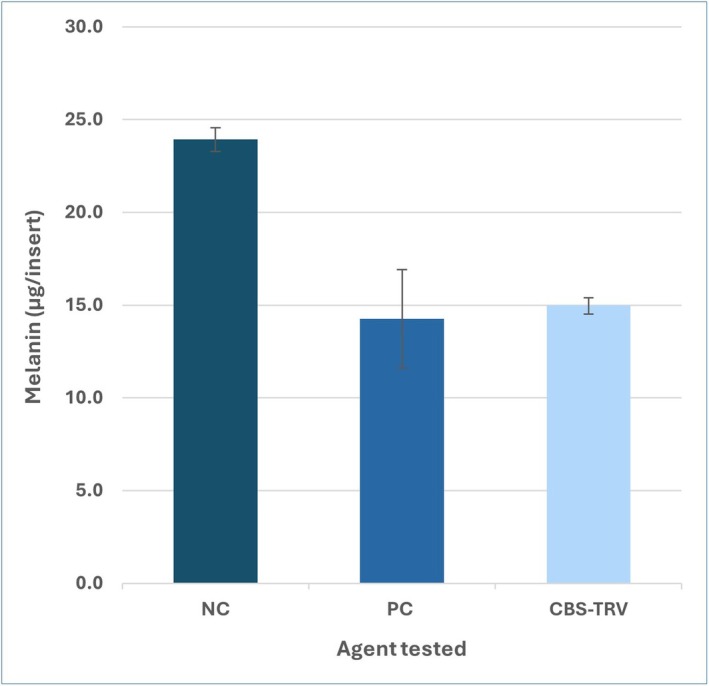
Melanin concentration in 3D tissues after 14 days of topical application of test products. CBS‐TRV had an effect comparable to the positive control (2% kojic acid). CBS‐TRV, complexion brightening serum; NC, negative control/untreated; PC, positive control/kojic acid.

In the Melanoderm assay, after 14 days, a 2 μL every other day topical application of CBS‐TRV was found to impede melanin production by 37.5%, which is comparable to the positive control (25 μL of 2% Kojic acid); see Figure 2. When compared to untreated tissues, there was no significant decrease (*p* < 0.05) in tissue viability for tissues treated with any of the test materials or dose volumes.

### 3.3 Clinical Trial

#### 3.3.1 Patient Population

A total of 23 subjects completed the study, with a mean age of 63.6 years (range: 32–73 years). As shown in the group demographics listed in Table 1, the majority of subjects were female and white or Caucasian. Fitzpatrick skin types I, II, III, V, and VI were represented.

**TABLE 1:** Subject demographics (*n* = 23).

Characteristic	*n* (%) or Mean ± SD	Range
Age (years)	63.6 ± 9.2	32–73 years
*Gender*		
Female	22 (95.7%)	
Male	1 (4.3%)	
*Ethnicity*		
Not Hispanic or Latino	23 (100%)	
*Race*		
Asian	3 (13.0%)	
Black or African American	3 (13.0%)	
White or Caucasian	17 (73.9%)	
*Fitzpatrick skin type*		
I	2 (8.7%)	
II	11 (47.8%)	
III	5 (21.7%)	
IV	0	
V	4 (17.4%)	
VI	1 (4.3%)	
*Fitzpatrick V–VI race subgroup*		
Asian	2 (8.7%)	
Black or African American	3 (13.0%)	

Abbreviation: SD, standard deviation.

#### 3.3.2 Efficacy

**FIGURE 3:**
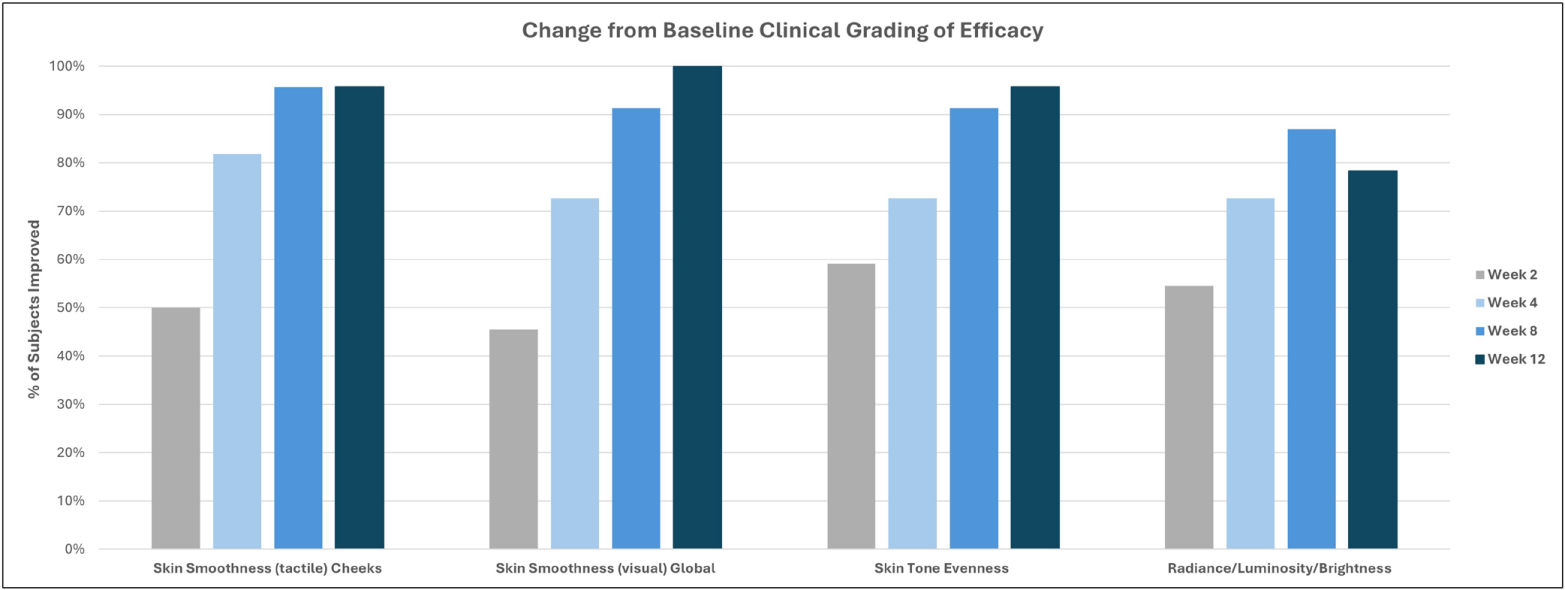
Change from baseline in clinical grading parameters in subjects treated with CBS‐TRV. Clinical grading was performed using the modified Griffiths 10‐point scale and included tactile assessment of skin smoothness on the cheeks, visual evaluation of overall (global) skin smoothness, skin tone evenness, and global radiance/luminosity/brightness.

Clinical grading change from baseline showed the majority of parameters improved significantly throughout the 12‐week course of the study, with a rapid onset of action apparent at week 2 (Figure 3).

**FIGURE 4:**
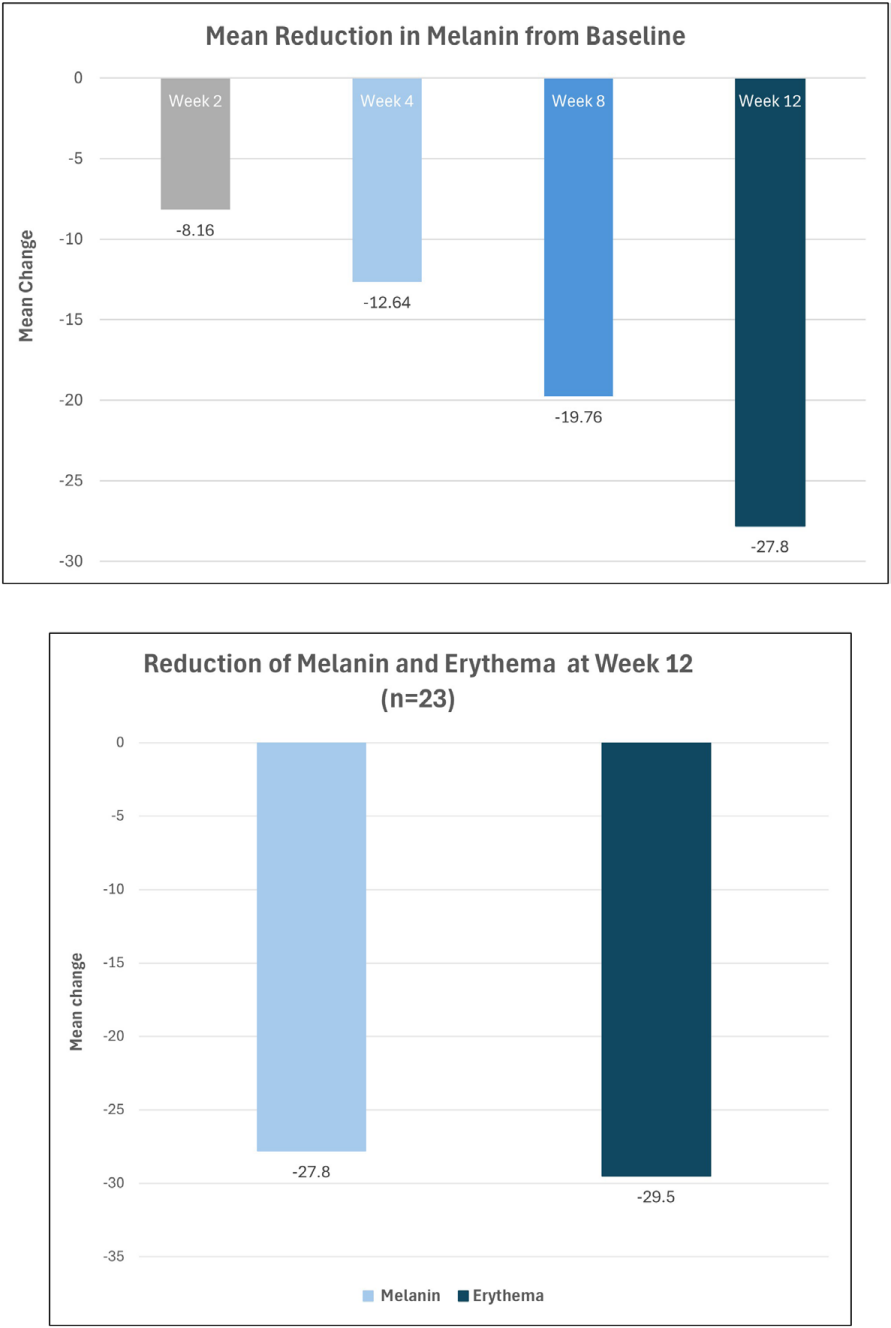
Mean reductions from baseline (mexameter). Top, mean change in melanin; bottom, reduction in melanin and erythema at week 12.

Mexameter measurements demonstrated statistically significant reductions in both melanin (−27.8 ± 36.9; *p* = 0.002) and erythema (−29.5 ± 54.2; *p* = 0.016) compared with baseline (Figure 4), indicating visible improvement in both pigmentation and erythema at week 12.

**FIGURE 5:**
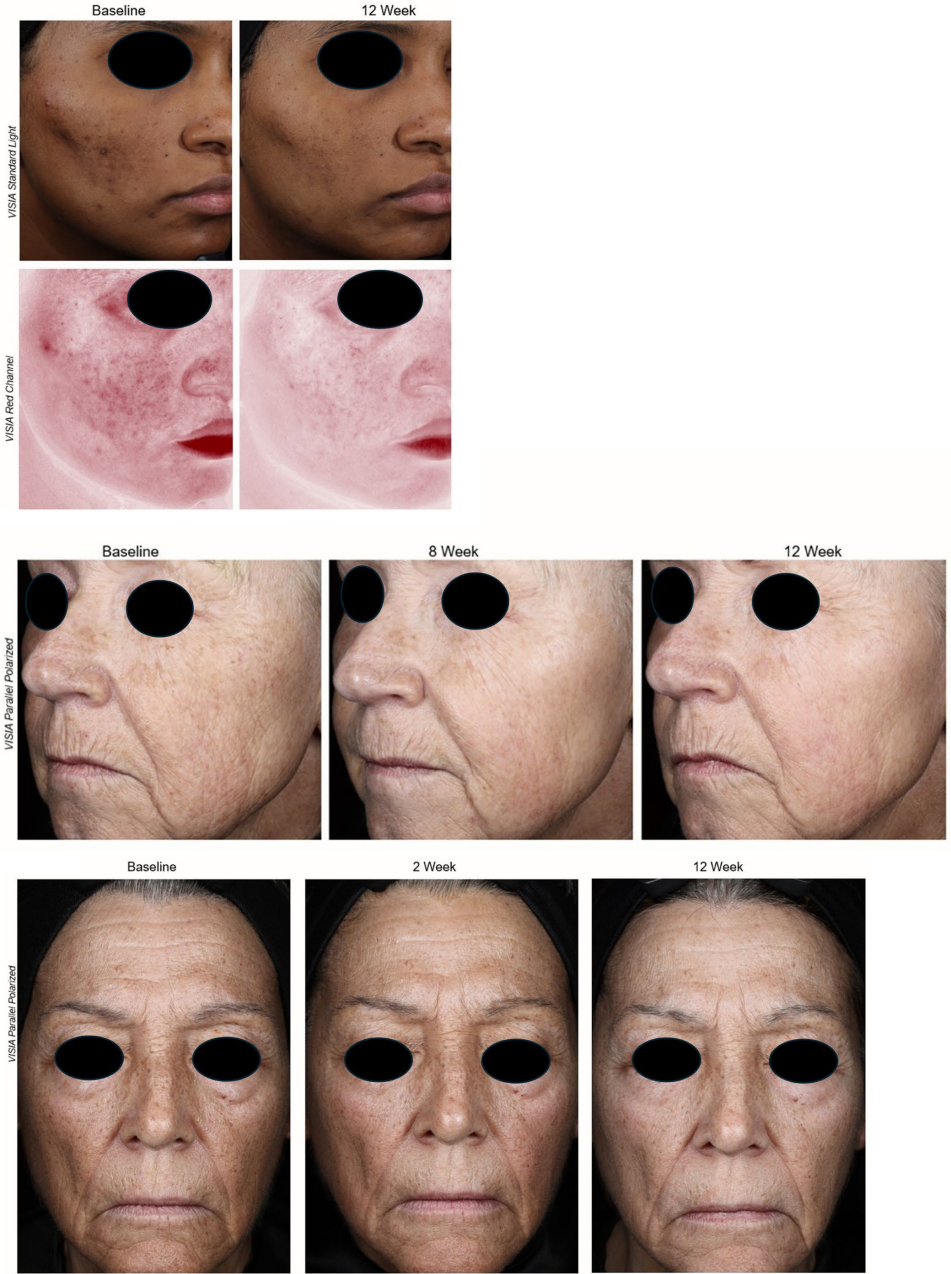
Standardized digital facial photographs of selected study subjects captured at baseline and through week 12 following treatment with CBS‐TRV. Images demonstrate visible improvement in facial hyperpigmentation over the treatment period. For some subjects, interim visits are shown to depict the progression of response.

Representative standardized digital facial photographs further illustrate visible improvement in facial hyperpigmentation over the 12‐week treatment period (Figure 5).

In the subject self‐assessment questionnaire, subjects indicated their dark spots and/or uneven skin tone made them feel uncomfortable and less confident at baseline. At week 12, there was a significant reduction in the subjects that reported discomfort and lack of confidence linked to skin tone than at baseline. Subjects reported favorable perceptions of smoother skin texture, brighter skin, reduced facial redness, more even skin tone, less visible discoloration and brown spots, improved pore size, and overall appearance. Subjects also reported liking the aesthetic qualities of the CBS‐TRV formulation.

#### 3.3.3 Safety/Tolerability

CBS‐TRV was safe and well tolerated during the study. Three non‐serious adverse events (AEs) were observed, two unrelated and one possibly related where mild skin irritation around the eyes was reported. The subject deviated from product usage instructions and inadvertently applied CBS‐TRV too close to the eyes.

## 4 Discussion

These studies demonstrate that a novel brightening serum with Tiered‐Release Vesicle delivery technology, termed CBS‐TRV, has a significant impact on skin tone and pigmentation. In vitro experiments using Melanoderm skin tissues indicated that CBS‐TRV applied for 2 weeks can reduce natural melanin production by at least 37.5% compared to untreated tissues. Furthermore, significant penetration of TXA into the epidermis was observed by Raman spectroscopy.

The 12‐week clinical study demonstrated statistically significant improvements in skin tone and pigmentation. There was substantial enhancement in overall skin appearance and smoothness. The clinical grading results were supported by statistically significant improvements in mexameter results. As demonstrated by self‐reported questionnaires, subjects also had a favorable perception of the overall appearance of facial skin and the formulation's aesthetic qualities.

To further explore the potential of CBS‐TRV in pigmentary disorders beyond dyschromia, a small controlled case series was performed in patients with melasma (*n* = 4). The study followed the same protocol as the dyschromia trial. After 12 weeks of treatment, average modified Melasma Area and Severity Index (mMASI) scores decreased from 8.0 at baseline to 2.0 at week 12 (≈75% reduction), with consistent improvement across all subjects. In addition to reductions in melasma severity, the treating physician noted improvements in redness, luminosity, and overall skin tone, which in some cases resembled the even complexion achieved after laser treatments. However, melasma associated with deeper pigmentation appeared resistant to topical treatment alone. Patients reported high satisfaction and tolerated the regimen well, with no adverse events observed. These results suggest that CBS‐TRV should be studied both as a stand‐alone regimen and in combination with active procedures and/or treatments to optimize outcomes.

We believe that certain active ingredients in CBS‐TRV (TXA, MI9, and linoleic acid from retinyl linoleate), effectively delivered by TRV technology, can serve to reduce the effects of UVB and other inflammatory factors on pigmentation, while the remaining ingredients (4BR, niacinamide, retinol from retinyl linoleate, and Vitamin C) function not only in a stimulated environment but also under steady state conditions. For many of these compounds, especially TXA, peptides, and Vitamin C, delivery into skin has long proven a major challenge, which can now be achieved with TRV technology. These findings continue to underscore the potential of TRVs to revolutionize skincare by effectively delivering bioactive molecules to targeted layers of the skin [3]. Furthermore, the preliminary melasma data complement the mechanistic findings of enhanced tranexamic acid penetration, MI‐9 receptor antagonism, and reduced melanogenesis in MelanoDerm models, underscoring the need for larger controlled trials involving patients with melasma. Overall study limitations included the open label design and the small number of subjects.

As more people move away from complex, multi‐step routines, they seek simple yet effective skincare solutions with compound effects. A multifunctional, well tolerated product promotes better compliance, ensuring consistent use, which leads to sustained results. By delivering both rapid and long‐term benefits, this product supports clinical efficacy and safety while aligning with the growing preference for simplicity in skincare.

## Author Contributions

S.K. and A.O. conducted the melasma case study research and contributed to data interpretation. A.M. and A.C.B. assisted with data interpretation. R.L., T.F., and S.O. drafted the manuscript and performed data review. All authors reviewed and approved the final version of the manuscript.

## Funding

This work was supported by Ourself.

## Ethics Statement

The authors confirm that the ethical policies of the journal, as noted on the journal's author guidelines page, have been adhered to and the appropriate ethical review committee approval has been received. IRB approval from Advarra IRB.

## Consent

All subjects provided written informed consent for their likeness to be used in educational materials, including publications such as medical journals.

## Conflicts of Interest

A.C.B. served as a consultant/speaker/investigator for Allergan/AbbVie, Arcutis, Avita Medical, Bausch Health, BiaCure, Biofrontera, Cutera, Cynosure, Cytrellis, Emblation, Estee Lauder, Jeisys, Lilly, Luminoma Diagnostics Limited, Medline, MiMEDx, Ourself/Glo Pharma, Novascan, Pfizer, R2 Technologies, RBC Consultants, Revance, Regeneron, Restore Biologics Holdings LLC, Suasion Group LLC, Sun Pharmaceutical Industries Inc., Theravant, Corp, and UCB Inc. A.O., S.K., and A.M. have served as consultants for Ourself. T.F., R.L., and S.O. are employees of Ourself.

## Data Availability

The data that support the findings of this study are available on request from the corresponding author. The data are not publicly available due to privacy or ethical restrictions.
